# Exploring the thoughts, emotions, and behaviours related to the self-management practices of adults with type 2 diabetes

**DOI:** 10.1177/20551029241278976

**Published:** 2024-09-08

**Authors:** Elné Visagie, Elmari Deacon, Rümando Kok

**Affiliations:** 1 56405 North-West University, COMPRES and University of Pretoria, South Africa; 2 56410 North-West University, Optentia, South Africa; 3 56410 North-West University, COMPRES, South Africa

**Keywords:** adults, behaviours, cognitive behaviour therapy, emotions, self-management, thoughts, type 2 diabetes

## Abstract

This qualitative research study explored the thoughts, emotions, and behaviours of adults aged between 35 and 45 who managed their type 2 diabetes effectively and adults who struggled with diabetes self-management in a South African setting. Semi-structured interviews were conducted with 17 adults who engaged in either successful self-management or who struggled with self-management. Effective management was characterised by an HbA1c level of 8% or lower. This group comprised of nine individuals. The participants who faced challenges with self-management had HbA1c levels ranging between 10% and 14%. This group consisted of eight participants. The data were analysed using inductive thematic analysis, and four main themes were identified: the emotional experience, prominent cognitions, practising acceptance and the mechanisms of behavioural change. These themes identified key determinants of individuals’ self-management practices and can contribute to providing information for future cognitive behaviour therapy interventions to be developed that target specific components to improve self-management practices.

## Introduction

Globally, diabetes has been recognised as a public health concern (Vlachou et al., 2022). Type 2 diabetes (T2DM) is viewed as a complex and multifarious metabolic syndrome due to the management challenges and several acute and chronic complications associated with the condition (Mayer-Davis et al., 2017). Aetiological risk factors include age, ethnicity, family history and lifestyle factors, such as increased obesity, dietary patterns, and sedentary lifestyles (Khan et al., 2020). It is estimated that 537 million adults live with diabetes, with T2DM accounting for approximately 90% of these cases (IDF Diabetes Atlas, 2021; Singer et al., 2022). In South Africa, the IDF (2021) estimates that one in nine adults has diabetes mellitus. The average age of onset is approximately 45 years for T2DM; however, there has been a rapid growth of T2DM diagnosis in individuals younger than 45, especially in developing countries within both urban and rural settings (Hu et al., 2021; Khan et al., 2020; Pheiffer et al., 2021).

The onset of T2DM is associated with an increased risk of developing diabetes-related complications and suboptimal adherence to management (Huo et al., 2018). Thus, the diagnosis of T2DM is accompanied by the realisation that the individual may face long-term complications, such as cardiovascular disease, diabetic retinopathy, and damage to several organs (Khan et al., 2020). The difficulties associated with T2DM self-management extend from a physiological to a psychological, behavioural and environmental realm (Hu et al., 2023). Initially, individuals may have to navigate an overload of information and critically examine the support and guidance provided by healthcare providers. Furthermore, they must adjust to a new medical care routine, which necessitates them to make behavioural adjustments (Hu et al., 2023). Behavioural challenges could include adherence to medication regimens, dietary modifications, regular physical activity, and monitoring blood glucose levels consistently (Tuobenyiere et al., 2023). Psychologically and emotionally, there are several changes that individuals face, such as the burden of self-care responsibilities, distress about diabetes-related complications, and feelings of frustration or guilt when unable to meet medical and self-management goals (Koochaksaraee et al., 2022). The diagnosis of T2DM also extends to the individual’s environment and social network. Individuals have to navigate social gatherings, cultural beliefs, socioeconomic status and support structures. These factors can either facilitate or hinder individuals’ ability to maintain optimal self-care practices (Tuobenyiere et al., 2023).

T2DM management is a complex experience filled with ambivalence, especially as adults are at a developmental stage where they balance several responsibilities, such as work, relationships, finances and family, which contest the demands of T2DM management (Lindh and Blomqvist, 2019). A diagnosis of T2DM requires individuals to accept responsibility to continuously navigate and address the management needs and demands required by this condition (Callan et al., 2022). It is a condition that necessitates adults to obtain an array of knowledge, skills, and self-confidence as they deal with most of the responsibilities and daily self-management tasks that are central to their disease management (Koochaksaraee et al., 2022). It has been evident that many adults struggle with self-management due to its demanding nature (Callan et al., 2022). Self-management is defined as active participation in managing one’s condition and is fundamental to the successful management of T2DM (Wang et al., 2018). Self-management encompasses different health-related activities and behaviours an individual undertakes to treat, control, and improve their health and well-being (Fiqri et al., 2022). Tasks can include medication adherence, adequate nutrition intake, adopting the required new behaviours in the context of T2DM, and emotional management (Adu et al., 2019). Effective self-management is typically associated with achieving and maintaining an HbA1c level of 7% or less (ElSayed et al., 2023). A less stringent HbA1C goal of 8% may also be agreed upon (Alqudah et al., 2019; ElSayed et al., 2023). Furthermore, it refers to the consistent application of behaviours and strategies to reduce the risk of complications and enhance the overall quality of life. These practices typically include adherence to medication, maintaining healthy dietary practices, engaging in regular physical exercise, monitoring blood glucose levels, and actively participating in diabetes education and self-care activities (Ahmad and Joshi, 2023). Conversely, poor diabetes management is often characterised by elevated HbA1c levels, typically above 9% or higher. Factors underpinning poor management may include irregular medication adherence, reduced or avoidance of medical appointments and reduced self-care practices.

It is apparent that the condition requires significant attention and careful consideration, and therefore, the management of the condition is something that only some adults commit to (Adu et al., 2019). It is apparent that there is a degree of diversity in the self-management of T2DM. The ability to effectively self-manage diabetes is influenced by various internal and external factors that can act as barriers or enablers. Despite large numbers of non-adherence, some individuals accept the inherent conflict of the diagnosis and practice effective self-management. Therefore, it is vital to explore how these individuals align self-management to their internal values to commit to the extensive lifestyle changes necessitated by the condition (Lindh and Blomqvist, 2019).

Understanding effective self-management is fundamental to comprehending individuals’ inner workings and how it enables them to commit to self-management within their social context (Fink et al., 2019; Winkley et al., 2020). Dealing with the diagnosis and navigating towards effective self-management requires an individual to tap into their cognitive beliefs about the condition, endure emotional experiences, and adjust behavioural routines (Van Smoorenburg et al., 2019). It requires individuals to work through and manage emotions, such as frustration, despair, and hopelessness regarding living with T2DM, interact with healthcare providers, navigate interpersonal relationships, and manage the emotional burden of having the condition (Van Smoorenburg et al., 2019). Furthermore, individuals with T2DM must navigate the fear of current and future complications while engaging in the decision-making process regarding managing the condition better and implementing these changes (Tolentino et al., 2022).

However, there is a need for more clarity regarding what thoughts, emotions, and behaviours serve as barriers to self-management and what serves as enablers (Lindh and Blomqvist, 2019). Cognitive behavioural therapy (CBT) can help to address cognitive, emotional, and practical barriers to T2DM management. CBT is based on the premise that individuals’ emotions and behaviours are influenced by their thoughts and perceptions of events (Beck, 1965). The interpretation of the event or situation influences how a person feels and reacts, not the situation itself (Beck, 1965; Fenn and Byrne, 2013). CBT is an umbrella term and encompasses various therapies and techniques that share core principles aimed at addressing cognitive and behavioural patterns contributing to psychological distress or maladaptive behaviours (Carona, 2023; Hayes and Hofmann, 2017). These therapies share foundational elements with CBT, such as identifying and modifying maladaptive thoughts and behaviours (Carona, 2023). However, they each have unique emphases and techniques tailored to address specific psychological disorders or challenges. The various approaches have proved effective across various populations and conditions, offering flexibility in treatment approaches based on individual needs and preferences (Carona, 2023; Hayes and Hofman, 2017).

The cognitive model, central to CBT, suggests that the behavioural changes individuals with T2DM may struggle with could be explained by their perception of T2DM and T2DM self-management (Fiqri et al., 2022). CBT interventions aim to facilitate the understanding of T2DM, explore the beliefs about self-management, practise acceptance, and build a repertoire of behavioural skills to manage T2DM effectively (Pan et al., 2020). Ultimately, CBT can be implemented to improve self-management by aiding individuals to identify the content of their thoughts, restructure negative thoughts regarding themselves and their self-management, reduce emotional distress, and foster constructive self-management behaviours (Callan et al., 2022).

Current research has indicated that CBT interventions can be effective in facilitating health outcomes of T2DM (Fiqri et al., 2022; Gulley et al., 2022; Vlachou et al., 2022). However, the majority of the studies focus on how CBT can improve psychological conditions, such as depression in individuals with T2DM (Abbas et al., 2023; Clarke et al., 2019; Cummings et al., 2019; Mansour et al., 2022; Mohamed et al., 2019) and does not focus solely on administering CBT for the improvement of self-management. Additionally, there is a lack of clarity about whether a specific CBT approach (e.g., traditional CBT, MBCBT, ACT, or DBT) or format (individual, group-based, or online) will be most effective and appropriate for individuals with T2DM (Fiqri et al., 2022; Yang et al., 2020).

Visagie et al. (2023) conducted a rapid review that identified scientific literature that specifically focused on CBT and self-management. Nine articles were identified, and all employed quantitative methods, thus highlighting the need for qualitative exploration of the subject matter.

However, before employing a CBT intervention, it is necessary to explore the relevant cognitive content and processes that need to be targeted, specifically for T2DM (Ayre et al., 2021). By exploring the internal dynamics through the view of the individual, who is at the centre of their self-management, pertinent thought and behavioural patterns that act as facilitators or barriers can inform which components need to be targeted to facilitate self-management (Fink et al., 2019). Therefore, there is a need to identify the specific thoughts, emotions, and behaviours displayed by adults who manage their diabetes well and those who struggle. This research can identify specific cognitive patterns, such as the positive thinking patterns and proactive problem-solving approaches of those who manage their diabetes successfully, as well as the challenging or maladaptive thoughts that hinder those who struggle. Furthermore, it can highlight emotional strategies that support effective self-management while also identifying emotional barriers that impede it. Obtaining this nuanced understanding can inform the development of tailored CBT approaches that enhance self-efficacy, emotional resilience, and sustained behaviour change for individuals struggling with self-management. Thus, this study set out to explore the thoughts, emotions, and behaviours of adults who manage their diabetes effectively and adults who struggle with diabetes self-management in a South African setting. It aimed to provide contextual examples for the development of CBT interventions that can provide culturally sensitive and contextually relevant content aimed at improving diabetes self-management outcomes.

## Methods

### Rationale for qualitative design

A qualitative design with a slight realist approach (Hammersley, 1992) was used. This approach acknowledges an external reality independent of personal beliefs and perceptions but underscores that an individual’s understanding of reality is mediated by subjective perceptions, interpretations, and social constructs (Emmel, 2013). The qualitative approach enabled the researcher to assess complex multi-component systems and create information that can bring social change to a current healthcare dilemma (Busetto et al., 2020). It facilitated the exploration and understanding of the mechanisms of change, how they work and for whom, why, and when they are implemented (Busetto et al., 2020).

### Participants and recruitment

Individuals who formed part of the Guidepost platform were recruited for the current study. The researchers utilised the two groups from the Guidepost platform: those who manage their diabetes well (HbA1c less than 7%) and those who show no improvement in management after the intervention, the high group (HbA1c 10%–14%). The groups and corresponding HbA1c levels are determined by Guidepost. Professor Segal, the co-founder of Guidepost, acted as the gatekeeper for this study. Purposive sampling was performed to identify and select participants who conformed to the following criteria:

Participants had to be between the ages of 35 and 45 years. The average age of diagnosis for T2DM is 45; however, individuals are frequently diagnosed between the ages of 40 to 45 (Sattar et al., 2019; Zoungas et al., 2014), with more cases being diagnosed in individuals between the ages of 20 to 39 years (Hu et al., 2021; Khan et al., 2020; Sattar et al., 2019). Therefore, the study aimed to focus on adults between the ages of 35 and 45.

To minimise the impact of adjusting to the condition and enhance the trustworthiness of the data obtained, participants had to have received a diagnosis of T2DM more than 1 year ago.

Participants had to form part of the Guidepost platform to promote homogeneity and reduce treatment variables.

Participants had to have maintained an HbA1c (average blood glucose level) of less than 8%, or between 10 to 14%, for the past year. Although the American College of Physicians (ACP) prescribes an HbA1c of <7%, findings by Smetana et al. (2019) and Qaseem et al. (2018) state that an HbA1c of 8% or less is effective for managing T2DM and proves beneficial for decreasing the development and advancement of microvascular and macrovascular complications. Therefore, the target of <8% was accepted to allow more participants to take part. Noteworthy is that only two participants in the lower HbA1c group had an HbA1c of >7%.

The researchers excluded individuals engaged in any form of psychotherapy as the psychotherapeutic process might have impacted their conceptualisation, thoughts, emotions, and behaviours related to self-management.

Guidepost identified a list of potential participants that met the inclusion and exclusion criteria. Participants received an information leaflet and could contact the researcher for further enquiries. The independent person screened potential participants who indicated an interest in the research project, and a subsequent date for the interview was set. An independent person obtained informed consent before the interview commenced. Data saturation was reached after the fifteenth interview as no new themes emerged. However, two more interviews were conducted to enhance the rigour of the data collection process. Thereafter, recruitment and data collection ceased. The final sample consisted of 17 adults represented by pseudonyms in Table 1. Nine participants were male, and eight participants were female. One of the participants’ home languages was isiZulu; another was Sesotho; five participants were Afrikaans-speaking, and ten were English-speaking. The participants had an average age of 40.65 years and an average HbA1c of 10.98% for the high HbA1c group and 6.38% for the HbA1c low group.

**Table 1.** Summary of participants’ demographics.

Pseudonym	Age	Gender	Ethnicity	Home language	Marital status	Year of diagnosis	Average HbA1c(%)
Participants with a high average HbA1c							
Mandla	39	Male	African	English	Married	2011	10,0
Siyanda	40	Male	African	English	Married	2004	14,0
Vanessa	35	Female	Coloured	Afrikaans	Married	2019	10,0
Nyakallo	42	Female	African	Sesotho	Married	2003	10,0
Lungani	42	Male	African	English	Single	2021	9,6
Latika	43	Female	Indian	English	Married	2007	11,2
Krisha	43	Female	Indian	English	Single	1996	11,4
Amahle	42	Female	African	isiZulu	Married	2009	11,6
Participants with a low average HbA1c							
Zayden	42	Male	Coloured	English	Married	2019	6,0
Advika	38	Female	Indian	English	Married	2020	7,6
Frans	42	Male	White	Afrikaans	Married	2007	6,1
Adrian	43	Male	White	Afrikaans	Married	2013	6,0
Eliana	45	Female	White	Afrikaans	Married	2016	6,0
Hannah	40	Female	White	English	Married	2008	5,6
Vasu	39	Male	Indian	English	Married	2021	6,7
Avinesh	37	Male	Indian	English	Married	2017	7,1
Joshua	39	Male	White	Afrikaans	Married	2003	6,3

### Ethical considerations

Ethical approval for the study was obtained from the Health and Research Ethics Committee (HREC) at the Potchefstroom North-West University campus, reference number NWU-00301-21-A1. During the COVID-19 pandemic, individuals with T2DM were considered high risk and the interviews were conducted via an encrypted online platform, Zoom, to ensure privacy and confidentiality. Participants were encouraged to be interviewed in a safe, confidential, and familiar place. The voluntary nature of participation was emphasised to the participants. Owing to the vulnerable nature of the participants (having a diagnosis of T2DM) and the possible sensitive content discussed during the interviews, measures, such as debriefing, were made available by a qualified healthcare professional, to ensure the comfort of participants. If further assistance was required, participants could be referred to a qualified psychologist who could conduct an online session.

A code was assigned to each participant. The consent forms and code list were stored separately. Quotations used in the research study were anonymised by providing pseudonyms to participants. The electronic data generated were password-protected, and hard copies were stored securely.

### Interview schedule

The semi-structured interviews lasted approximately 45 min and adhered to an interview agenda. The questions revolved around the participants’ journey since being diagnosed with diabetes, including challenges and successes in self-management. They discussed the emotional and cognitive aspects of these experiences, such as their thoughts and feelings related to difficulties and victories. Questions also addressed specific behaviours that either hindered or improved their self-management and explored the range of emotions experienced throughout their journey. Some of the questions included were:

What have been the challenges with regards to self-management?

What thoughts are related to this, emotional reactions, and how do you respond to this?

What has been good with regards to self-management, or what victories have you experienced with your self-management tasks

How did this make you feel? What did you think? (Follow up question)

What are the negative thoughts associated with your self-management?

How do you think about the self-management tasks, the self, and your abilities?

What behaviours have hindered your self-management?

What thoughts are linked to these behaviours? (Follow up question)

What behaviours have improved your self-management?

### Data analysis

The recorded interviews were transcribed verbatim by the researcher to allow data immersion. The researcher and other members of the research team ensured the accuracy of transcriptions by checking the recordings of the interviews. The researcher used Atlas.ti to organise the data and facilitate data coding. The data was analysed by means of thematic analysis, as set out by Braun and Clarke’s (2006, 2013) six-step process. These steps included (1) data immersion, (2) developing initial codes, (3) identifying themes, (4) reviewing themes, (5) defining the themes, and (6) reporting the results. Data familiarisation and immersion occurred as the researcher continuously reviewed and read the interview transcripts to identify initial codes. A co-coder independently analysed the data to enhance the rigour and reliability of the findings (Braun and Clarke, 2013). The researcher and co-coder independently coded to the dataset. Once codes were identified, the researcher and co-coder met regularly to compare codes, resolve discrepancies and identify consistently present codes. Inductive reasoning was employed to facilitate exploration and to identify the dataset’s codes, categories, and themes. This aligns with research done by Braun and Clarke (2013), who elucidate that the engagement of multiple coders can mitigate idiosyncratic interpretations, thereby facilitating a form of triangulation during the formation of themes. The coded data was synthesised and developed into coherent themes and sub-themes. Discussions took place with the three researchers involved to reach a shared understanding and credibility of the findings. Subsequently, the co-coder interpreted the themes in the context of the research question and verified that the findings were reflective of the dataset and understood within the context. Credibility and trustworthiness were ensured through triangulation, prolonged engagement, reflexivity, and member checking (Lincoln and Guba, 1985). During data generation, the study employed investigator triangulation as discussions were held, and the themes were reviewed by three independent reviewers to ensure cogency and reliability and to enhance the trustworthiness of the findings and how it was interpreted (Connelly, 2016).

Credibility was achieved through gatekeepers at Guidepost, who identified participants that could make meaningful contributions to the study. The researchers engaged in prolonged engagement and reflexivity through data immersion and reflective journals. There was a strong cognitive behavioural therapy orientation from the researchers, and they were continually conscious of how their dogmas could influence the analysis process. To ensure the confirmability of the results, the quotations used were grounded in the dataset and not based on conjectures and assumptions. Three researchers independently analysed the data and, through discussions, reached a consensus regarding the reliability and saturation of the data. Transferability was attained by providing detailed descriptions of the results and contextualising the research process and methodology.

## Results

In exploring the thoughts, emotions, and behaviours of adults with T2DM, four main themes were identified, as indicated in Table 2, namely (1) the emotional experience, (2) prominent cognitions, (3) practising acceptance, and (4) mechanisms of behavioural change. These themes provided insight into how adults who managed their T2DM effectively (low HbA1c group) and those who struggled (high HbA1c group) conceptualise the self-management of their T2DM, the emotional processes that they had to work through, the behavioural techniques they used to improve management, and the challenges they continuously face.

**Table 2.** Themes and subthemes.

Main theme	Subtheme
Theme 1: The emotional experience	1.1. Initial emotions
	1.2. Dealing with challenging emotions
	1.3. Generating positive emotions (victory promotes adherence)
	1.4. The role of stress
Theme 2: Prominent cognitions	2.1. The ‘why me’ thought
	2.2. Negative thoughts regarding the condition
	2.3. Cognitive technique: Identifying the controllable
Theme 3: Practicing acceptance	
Theme 4: Mechanisms of behavioural change	4.1. Tapping into motivation
	4.2. Utilising resources
	4.3 The process of behavioural change
	4.4. The challenges of self-management

The 17 interviews were thematically analysed together to identify overarching themes and patterns that may not have been apparent when separating the groups. Table 3, A Summary of Differences Between Low and High HbA1C Group, summarises the differences between the two groups. In contrast, the themes discussed in the results address the data as a whole. This approach ensured that the results delineated the full spectrum of experiences and perspectives, providing a richer and more nuanced understanding of the factors influencing diabetes management. The themes discussed revealed the commonalities and distinctions within the two groups and allowed the research to build a framework for understanding the diverse challenges and strategies related to self-management.

**Table 3.** A summary of differences between low and high HbA1C group.

Theme 1: The emotional experience (The emotional component)	
Low HbA1c group	High HbA1c group
**Initial emotions:**	**Initial emotions:**
Anxiety, shock, denial	Anxiety, shock, denial
**Challenging emotions:**	**Challenging emotions:**
Guilt, frustration	Guilt, frustration, hopelessness, fear
**Positive emotions:**	**Negative emotions:**
Generation of positive emotions	Remaining stagnant in negative emotional states
Motivation, self-efficacy	Depression, helplessness

Theme 2: Prominent cognitions (The cognitive component)	
Low HbA1c group	High HbA1c group
**Cognitive themes:**	**Cognitive themes:**
“Why me” thoughts	“Why me” thoughts
Reframing negative thoughts	Negative perceptions
**Cognitive techniques:**	Feeling trapped by the condition
Identifying what is controllable	**Cognitive challenges:**
Goal-setting	Difficulty reframing negative thoughts

Theme 3: Acceptance	
Low HbA1c	High HbA1c group
**Engagement with acceptance:**	**Engagement with acceptance:**
Comprehensive and ongoing engagement	The approach appeared superficial and lacked immersion
**Recognition of diagnosis**	**Recognition of diagnosis**
Acknowledged diagnosis and necessity of adjustments	Some exhibited avoidance and denial of diagnosis
**Perception of T2DM**	**Perception of T2DM**
Embraced the condition as a chronic illness	Neglected seriousness of the condition, living “normally.”
**Impact on self-management**	**Impact on self-management**
Facilitated positive changes in self-management	Hindered the ability to manage the condition effectively

Theme 4: Mechanisms of behavioural change (The behavioural component)	
Low HbA1c group	High HbA1c group
**Proactive behaviours:**	**Reactive behaviours:**
Seeking information	Struggling with adherence
Experimenting with lifestyle changes	Relapse into old habits
**Adaptive strategies:**	**Maladaptive responses:**
Utilising social support and internal resources	Coping through unhealthy behaviours
Setting achievable goals	Resistance to change

### Theme 1: The emotional experience

#### Initial emotions

The diagnosis of a chronic condition that requires lifelong management evoked several intense emotions in the participants from both groups. Participants experienced distress at the time of diagnosis with emotions ranging from anxiety, shock, anger, or denial. Many participants refused to believe the diagnosis due to the restrictive and uncomfortable nature of the condition, in addition to the lifestyle changes it required. Denial was a strong response, and several participants did not initially think they had T2DM; Frans (male, 42, HbA1c 6,1%) noted the denial, ‘*I do not believe it; how could this type of thing happen to me?’*

Participants struggled with accepting the diagnosis (discussed under Theme 3) and had to work through several unpleasant emotions. Initially, many participants opted to avoid reality and accept the truth. This was evident in both groups. Hannah (female, 40, HbA1c 5,6%) stated, ‘*I’ve gone through stages where I think the doctors are wrong; I don’t have it*.’ This was also partly due to the absence of significant physical symptoms, as Joshua (male, 39, HbA1c 6,3%) mentioned, ‘*I didn’t think about it often because I am not feeling bad (physically); I am unsure whether the blood results are correct.*’

Once the realisation started, strong emotional reactions, such as sadness, were evoked when participants received the diagnosis. Feelings of anger and fear accompanied this due to the uncertainty of the disease and the complete lifestyle change required. Joshua (male, 39, HbA1c 6,3%) said, ‘*You are angry, and you are unsure; it is a total change of everything in your life*.’ It was a continuous emotional rollercoaster as participants grappled with the diagnoses and had to deal with the required behavioural adjustment. A range of emotions was triggered when an individual faced a life-altering condition, and all the participants experienced several challenging emotions when faced with the diagnosis.

#### Dealing with challenging emotions

The emotional repertoire throughout the course of self-management consisted of intense feelings, such as guilt, frustration, hopelessness, and fear. All the participants reported continuously dealing with feelings of guilt and frustration. However, participants of the high HbA1c group most prominently conveyed feelings of hopelessness and fear.

Participants from both groups experienced guilt when they were conflicted about doing something they should not have done. The guilt that most participants experienced revolved around having done this to themselves (causing them to develop T2DM) and not adhering to management plans (such as overindulging). Mandla (male, 39, HbA1c 10,0%) noted the feeling of guilt, ‘*you feel guilty that you’ve caused this for yourself because you’ve done things that you shouldn’t have.’*

Guilt was evident when the participants deviated from their usual dietary patterns or self-management routine; Hannah (female, 40, HbA1c 5,6%) mentioned, ‘*You beat yourself up; you shouldn’t have let yourself go.’*

All participants reported feeling frustrated at some point due to self-management challenges. Frustration was evident when participants were not experiencing the desired results. Frustration is a complex emotional reaction, and it is seldom felt in isolation; participants’ emotions often escalated as they had to deal with an array of complex emotions continuously. Vasu (male, 39, HbA1c 6,7%) continued, *‘It’s frustration, and it is confusion and disappointment that no matter what I’m doing, I’m unable to achieve the goal*.’

The participants from the high HbA1c group conveyed feelings of fear, helplessness, and hopelessness. Feelings of hopelessness and helplessness were echoed in thoughts as ‘what is the whole point of this’ (further discussed under Theme 2), as Amahle (female, 42, HbA1c 11,6%) described, ‘*The everyday becomes what’s the point? You get to ask yourself, my whole life, I’ve done the right thing, but things still go wrong, and then it becomes, why should I even bother eating right? Because it really doesn’t matter.’* The intense feelings of sadness, hopelessness, and helplessness led to depressive feelings for some participants in the high HbA1c group, such as Nyakallo (female, 42, HbA1c 10,0%) who said, ‘*I was depressed because I became a lazy person, always feeling like I can sleep and the things that I was normally doing that made me happy, it didn’t matter anymore.’*

The participants’ emotional experiences were influenced by how they interpreted and made sense of their experiences, contributing to their various emotions. The beliefs underlying the emotional experience are discussed further under Theme 2.

#### Generating positive emotions (victory promotes adherence)

The self-management process entailed an array of emotional experiences, and despite the protuberant challenging emotions, participants were able to generate positive emotions through seeing positive health-related results. Experiencing self-management victories and achieving self-management targets generated a range of positive emotions and encouraged participants to stick to self-management plans. This was evident in most low and high HbA1c group participants. Vasu (male, 39, HbA1c 6,7%) stated ‘*What I’m doing is actually making a difference in my life and improving the quality of my life as well, and to see the improvements, I was ecstatic.’*

Experiencing these victories promoted a sense of motivation to keep to self-management plans. Still, as both groups experienced this, these positive emotions were not enough to sustain adherence to self-management (as discussed under Theme 4), as emotions are transient, and participants appeared to require a range of regulation skills, support, and resilience to sustain effective self-management.

#### The role of stress

Throughout T2DM self-management, stress was a prominent mental state that participants from both the high and low groups experienced. The participants mentioned internal and external sources of stress and emphasised the need for a plan to decrease their stress levels. Stress was entwined with several emotional responses and contributed to a negative emotional space. Participants in the high group found it difficult to navigate at times; Amahle (female, 42, HbA1c 11,6%) explained the compounding nature of T2DM-related stressors, ‘*when we are stressed, and things are not going your way, for me, it becomes a, I’ve done everything that I’m supposed to do. Why is this happening? And it’s very easy to feel sorry for yourself.’*

### Theme 2: Prominent cognitions

#### The ‘why me’ thought

Individuals who engaged in self-management conceptualised their T2DM in different ways and, therefore, experienced an array of negative and positive thoughts towards the condition. Both groups reported that they experienced the ‘why’ thought. Participants found it challenging to understand why they specifically developed T2DM and had to deal with the physical and emotional complications, as Zayden (male, 42, HbA1c 6,0%) stated, ‘*I was asking why? Why is this happening now? Why all of a sudden? Why not give me a heads-up that this is coming?’* Thoughts of ‘why’ progressed to ‘why me’, as participants grappled with the reality of their diagnosis.

Accompanying the ‘why me’ thoughts were ‘why me and not someone else’, which was especially prominent in the high HbA1c group participants. Mandla (male, 39, HbA1c 10,0%) expressed, ‘*I come from a family of seven kids, and I just don’t understand, out of all of them, it just chose me’*.

The ‘why me’ thought prompted additional thoughts, such as ‘What did I do wrong?’ These underpinned emotions of guilt and shame (Theme 1). Advika (female, 38, 7,6%) highlighted this by saying, ‘*What have I done wrong for me to have this chronic illness?’*

The thought of ‘why do I have to have this disease’ was apparent throughout self-management and was not one that only appeared initially. It is one that participants from both groups had to deal with and work through continuously. Vasu (male, 39, HbA1c 6,7%) echoed these thoughts of continually having to think of self-management-related behaviours, ‘*I’m going to have to keep on taking medication, and I got to keep on doing A, B, C, D in order to manage this*.’

#### Negative thoughts regarding the condition

Initially, participants from both groups viewed their diagnosis as a death sentence. The emotional impact of the diagnosis, in addition to the possible health-related complications, triggered thoughts such as ‘*my life is over’* as expressed by Mandla (male, 39, HbA1c 10,0%). Similarly, Joshua (male, 39, HbA1c 6,3%) expressed, ‘*Let’s ride the wave because we are going to die soon.’* It was apparent that participants in the low HbA1c group engaged in reframing their thought processes and changing their beliefs about self-management. Vasu (male, 39, HbA1c 6,7%) highlighted this shift by explaining, ‘I*t’s not a death sentence; it is a life sentence; I can change my behaviour and get better results.’* Reframing negative thoughts was not mentioned by the high HbA1c group.

Negative thoughts were expressed mainly by the participants in the high HbA1c group who struggled with self-management. Negative thoughts were not absent in the low group; however, there was less emphasis on these thoughts, as Hannah (female, 40, HbA1c 5,6%) mentioned, ‘*It’s okay. It’s going to be okay; it’s not as bad as you think it’s going to be; it’s not all doom and gloom*.’ Participants in the high HbA1c group expressed difficulty with thoughts such as ‘*I am not like others’* and ‘*I am restricted’*. This was a large area of contention for participants in the high HbA1c group, with several mentioning that it was challenging to realise and be reminded that they cannot do what others can. Latika (female, 43, HbA1c 11,2%) shared, ‘*I have friends that can go partying the whole night on, drinking, eating and whatever, but there’s no way I can. I’ll end up sick or in hospital.*’

Another prominent cognition experienced by individuals in the high group was *‘I am trapped/restricted’,* highlighted by Nyakallo (female, 42, HbA1c 10,0%). The restrictions and limitations that accompanied the diagnosis of T2DM triggered thoughts about being trapped and limited by the participants in the high HbA1c. These thoughts constructed a negative belief about self-management and contributed to the difficulty of managing T2DM.

#### Cognitive technique: Identifying the controllable

Participants in the low HbA1c group refocused their attention away from the negative thoughts surrounding the condition and identified what they could change. Advika (female, 38, 7,6%) mentioned, ‘*I’m only focused on the ones that I can control, the ones that I cannot control, I cannot focus on because I wouldn’t win this battle.’* All the low HbA1c group participants used the cognitive strategy of identifying what was in their control. By identifying what they could control, the participants were able to regulate their emotions, which promoted improved decision-making. This provided a sense of self-efficacy as they were the agents of change. The areas that participants could control were related to meal planning and preparation, being prepared for changes in routine, and gaining knowledge. Eliana (female, 45, HbA1c 6,0%) identified meal planning as controllable, stating, ‘*During the weekend, I plan meals for the following week and ensure that all the ingredients are there.’* Vasu (male, 39, HbA1c 6,7%) highlighted the need to be prepared, ‘*I don’t go anywhere without something being in the car, a sweet or something that I can give me a boost if I need it.’* Zayden (male, 42, HbA1c 6,0%) explained how he used reading labels to gain knowledge, ‘*I would turn products around, and I would look what the contents are in them. I still do this*.’

### Theme 3: Practicing acceptance

The process of acceptance was demonstrated in the emotions (Theme 1) and thoughts (Theme 2) that the participants experienced and had to work through. Although most participants mentioned the role of acceptance, the participants from the low HbA1c group appeared to have engaged more comprehensively with this process. In contrast, individuals from the high HbA1c group merely acknowledged the need for acceptance. Joshua (male, 39, HbA1c 6,3%) highlighted the complex process of acceptance, *‘I do not want to delve into what is really wrong and how this type 2 diabetes really works because I have an aversion towards what happens to you.’* He continued, ‘*Then you get to the point where you accept, these are the risks, this is how I need to manage the risks, what do I need to manage those risks, and okay, there are definite lifestyle choices that need to be made.’*

Participants in the low HbA1c group demonstrated that acceptance was a multifaceted and continuous process. One had to accept the diagnosis, as well as the various changes and adjustments that accompanied a diagnosis of T2DM. Advika (female, 38, 7,6%) explained this process, ‘*I actually accepted that I need to adjust to the new life, so I need to accept that I am now classified as being diabetic as having a chronic illness*.’ It was not a comfortable process; however, as the participants accepted their diagnosis, there were positive changes to self-management, and it facilitated the behavioural changes needed for effective self-management (highlighted under Theme 4).

From the high HbA1c group, it appeared that the process of acceptance lacked immersion. For several participants in the high HbA1c group, the lack of engagement with the diagnosis and wrestling with what this entailed appeared to contribute to not managing the condition effectively. Lungani (male, 42, HbA1c 9,6%) stated, ‘*I don’t even motivate myself. I don’t even think of diabetes. There’s no word that it is in my vocabulary.’* Furthermore, there appeared to be a lack of responsibility and avoidance of the seriousness of the condition; Lungani (male, 42, HbA1c 9,6%) explained, ‘*I’m living dangerously. After Covid, when it had me, I never go to take a vaccine; I usually am not wearing masks, sometimes in public. I don’t think of those things; I just live my normal life.’* Acceptance was crucial to implementing the changes required to effectively self-manage T2DM, and how that process of acceptance was engaged in influenced how an individual approached and maintained their self-management.

### Theme 4: Mechanisms of behavioural change

Ultimately, self-management warrants individuals making behavioural changes to their lifestyles. The process of self-management is not linear, and participants had to deal with their thoughts, emotions, and behavioural adjustments concurrently. However, it was apparent that the cognitive appraisal of the condition, the emotional association, and the level of acceptance either enabled or hindered participants to commit fully to behavioural changes. Behavioural changes appeared to progress from why I need to self-manage (Theme 4.1: Tapping into motivation) to what I need to do (Theme 4.2: Utilising resources) to how I do it (Theme 4.3: The process of behavioural change. These behavioural adjustments were most prominent in the low HbA1c group, with the participants in the high HbA1c group reporting several challenges (Theme 4.4).

#### Tapping into motivation

Participants had to delve into their reasons for adhering to their self-management plans. Self-management of T2DM was demanding and, due to its chronic nature, something that participants had to engage in constantly. Therefore, the participants needed to find something they believed in to promote adherence to their self-management plans.

Families, specifically children, proved to be a primary source of motivation for participants of both groups to improve their self-management. For some participants of the low HbA1c group, their children were important sources of motivation; however, many of the participants of the low HbA1c group had health-related aspirations that served as motivation, as displayed by Eliana’s (female, 45, HbA1c 6,0%) comment, ‘*I’d want to be healthier at 50 than I was at 45*.’

Participants had various goals and techniques that generated motivation, and the most important appeared to be finding what drives you; Krisha (female, 43, HbA1c 11,4%) said, ‘*You have to have a purpose as to why you want to continue. What do you want to do this for? Because that’s going to be what’s going to drive you every day.’*

Motivation can be fleeting, as displayed in Subtheme 4.4; therefore, participants had to use a combination of factors, such as motivation and internal and external resources, to promote improved self-management.

#### Utilising resources

The participants identified several resources that helped them come to terms with the diagnosis and adjustment to self-management. The prominent resources used by the participants from both groups were social support and obtaining information from various sources. Social support was offered by family, friends, healthcare professionals, and participants disclosing their diagnosis. Initially, social support was offered in terms of emotional support. Families and friends provided emotional support as the participants grappled with and accepted the diagnosis and associated adjustments. Joshua (male, 39, HbA1c 6,3%) explained, *‘He (friend) understands and knows what the perils are; he made it a lot easier for me. He helped me to become comfortable in a place where you are not fully at ease yet.’* As the participants adjusted to their routines, families adapted and offered tangible support in terms of self-management. Amahle (female, 42, HbA1c 11,6%) highlighted the important role of social support, *‘My fiancé started cooking the right food and started checking and making sure that I changed.’* Having people around who accepted the condition and offered emotional and tangible support contributed to improved self-management and, most importantly, generated a feeling of not having to face this intimidating task alone.

Informational support was mainly obtained through sharing knowledge with peers and engaging with various healthcare professionals. The difference between the two groups was highlighted in the actions of the low HbA1c group. These participants used themselves as a resource to obtain information beyond what was received from healthcare practitioners. This contributed to effective management, as the participants accepted the responsibility to supplement what was already known. The participants in the low HbA1c group facilitated their own learning and did much self-directed research in addition to the information obtained from healthcare professionals. Eliana (female, 45, HbA1c 6,0%) highlighted this process, ‘*I did a lot of reading, and I read a lot of recipe books and specifically recipes for diabetes, that did not only provide the instructions, but that explains why and how.’*

#### The process of behavioural change

Accepting the diagnosis and taking responsibility for self-management underpinned the behavioural process, which was evident in participants in the low HbA1C group (Theme 3) who engaged in behavioural change. Behavioural change was a continuous process that the participants in the low HbA1c group continually refined.

The behavioural change process started with developing an awareness of the body. Participants in the low HbA1c group became familiar with different physiological cues from their bodies, which indicated their blood sugar levels. Frans (male, 42, HbA1c 6,1%) explained, ‘*You have to listen to your body. I can basically feel it in my body when my sugar levels start getting high or if I go into a hypo. When I listen to my body, I can say whether I can eat something or I should rather not eat something, or I urgently need something sweet. I can work around it*.’

By knowing and being in tune with their body, the participants could engage in corrective action, felt more in control, and had a sense of security in knowing when to adjust or refrain from certain food.

As these participants felt more comfortable with physiological cues, the participants from the low HbA1c group started to experiment with different foods to see how their bodies reacted. They experimented with their readings to adjust their diets and lifestyle and ultimately improve their self-management. Vasu (male, 39, HbA1c 6,7%) explained the approach; ‘*I started researching what foods spike my blood sugar and what food with trial and error, and what food doesn’t.* For some participants, this involved using a diabetes monitor or writing down readings after having certain types of food.

These experiments enabled the participants to gain more knowledge (as discussed under Theme 4.2), make more informed decisions, and facilitated a sense of responsibility. Eliana (female, 45, HbA1c 6,0%) clarified*, ‘It really changes one’s behaviour as you immediately see when you were naughty like when I eat ice cream, it instantly shows you what you are doing, and it really changes your behaviour.’*

Participants in the low HbA1c group identified healthier alternatives that would not negatively affect their readings and, ultimately, their health. By identifying alternatives, the participants did not feel deprived and viewed their dietary plans as satisfactory and still appetising. Hannah (female, 40, HbA1c 5,6%) mentioned, ‘*I would find a different type of brand of chocolate. Something that’s more in the space that we would be allowed to eat in, and then I’ll just have a block, and then it helped.’*

It was noteworthy that these individuals from the low HbA1c group still experienced cravings; as Frans (male, 42, HbA1c 6,1%) stated, ‘*You still feel like eating, but instead of eating a chocolate, you eat a packet of peanuts*,’ but explored alternatives that do not aggravate their blood sugar levels. At times it was about making the healthier choice or identifying the healthier alternative; as Zayden (male, 42, HbA1c 6,0%) explained, ‘*Let’s take KFC,* for example*. So, would I do a wrap versus three pieces of chicken? I’d rather do the wrap.’* It was apparent that making the healthier choice led to a pattern of more considered eating and a more nutritious diet, which was essential for effective T2DM management.

#### The challenges of self-management

Throughout the process of behavioural change, all the participants experienced setbacks and challenges regarding their self-management. The low HbA1c group participants normalised slipping up and having a few off days. It was noticeable that these participants spent less time dwelling on these setbacks, which did not allow them to become part of the norm. Hannah (female, 40, HbA1c 5,6%) explained the response to a slip-up, ‘*It’s just like I’ve had something, okay, now we can move along.’*

Within the high HbA1c group, the participants found these setbacks challenging and tended to slip back into old patterns. Amahle (female, 42, HbA1c 11,6%) explained how unforeseen circumstances contribute to this, ‘*So, there are always things, you get into a routine for two, 3 weeks, then the kids are sick for a week, and then I’m sick the week after. So that’s 2 weeks out of your routine, and then it takes you time to get back into it.’*

It appeared that sticking to the routine was challenging, translating into participants making a conscious decision not to adhere to their management plan. This was partly due to participants’ relationship with food as Adrian (male, 43, HbA1c 6,0%) shared, *‘Sometimes eating is an emotional crux for me*.’T2DM placed great emphasis on diet and consuming certain food groups. Several participants from the high HbA1c group found this challenging, as food formed part of their coping mechanisms; as Amahle (female, 42, HbA1c 11,6%) explained, *‘You chose comfort food, and most of the time, comfort food is not healthy.’*

The themes identified reveal distinct emotional, cognitive, and behavioural dynamics associated with the self-management of T2DM within the groups. Table 3 summarises the main differences in relation to each theme as displayed by the low and high HbA1c groups.

## Discussion

This study aimed to explore the thoughts, emotions, and behaviours of adults who managed their T2DM effectively and adults who struggled with diabetes self-management in a South African setting. The main themes supported these findings and included (1) the emotional experience, (2) prominent cognitions, (3) practising acceptance and (4) the mechanisms of behavioural change. These themes suggest that CBT interventions tailored for T2DM should focus on several key areas to enhance self-management and provide insight into which emotional, cognitive, and behavioural barriers need to be targeted in those struggling with self-management. Furthermore, those who manage diabetes more effectively provided insight into the content of their thoughts, emotional strategies, and behavioural approaches that could be incorporated into future CBT interventions for self-management.

### Theme 1: The emotional experience

At the cornerstone of self-management was the notion that how individuals felt about their condition affected their behavioural responses and influenced cognitive appraisals, as delineated by Theme 1. The experience of receiving a diagnosis of T2DM is inherently laden with various emotional reactions, encompassing feelings of denial, anger, fear, and sadness. Acknowledging and expressing these challenging emotions during the initial phase of diagnosis is necessary, as it allows individuals to confront the reality of their condition and process the accompanying psychological discomfort. However, it is equally important for individuals to cultivate a sense of positive emotions as they navigate the diagnosis and adjustment. Participants in the low HbA1c group reported fostering positive emotions, which facilitated adaptive coping strategies and engendered a greater sense of agency and control over one’s health outcomes. This is important to consider when developing a CBT intervention targeting self-management. Visagie et al. (2023) reported that CBT interventions should focus on helping patients acknowledge and process negative emotions, cultivate positive feelings, and reduce rumination.

This was noticeably absent within the high HbA1c group, and participants tended to ruminate on feelings of fear and helplessness. Research has shown that sustained exposure to negative affectivity can negatively impact self-management practices and diminish overall quality of life (Kalra et al., 2018; McClain and Buchman, 2012). Overwhelming emotional demands diminish the resources required for everyday self-management, which contributes to unsatisfactory adherence (Wierenga et al., 2017).

The need to improve self-management out of fear generated negative emotions, as displayed by the high HbA1c group, whereas self-management to improve health was facilitated by a sense of encouragement, as presented by the low HbA1c group. Research suggests that fear does not effectively motivate change and does not lead to sustained behavioural changes (Walker et al., 2022). Comparatively, autonomous sources of motivation promoted longstanding behavioural change (Walker et al., 2022), as demonstrated by the low HbA1c group, who used their self-management victories to encourage adherence. Fink et al. (2019) found that experiencing self-management-related successes aided individuals in developing a sense of self-efficacy, which generated positive emotions and has been linked to adopting new behavioural self-management strategies. Thus, strategies that promote fostering intrinsic motivation through value-based goal setting and reinforcing small successes should be introduced in a CBT-based intervention to enhance self-efficacy and empower individuals in their self-management efforts. This aligns with research suggesting that positive emotions and autonomous motivation promote sustained behavioural change (Fink et al., 2019; Kalra et al., 2018; Walker et al., 2022).

### Theme 2: Prominent cognitions

The study highlighted the bidirectional relationship between emotions and cognitions, as their cognitive appraisals of self-management affected the participants’ emotional experiences. Both groups’ conceptualisation of their T2DM was initially founded on negative thoughts. However, after the diagnosis, these thoughts’ impact on self-management was related to the individual’s appraisal and restructuring of the thoughts.

As displayed in the high HbA1c group, negative cognitive appraisal indicated a stoical view and generated a sense of helplessness. The negative interpretation of a condition promoted greater doubt about the potential control over the condition and, therefore, decreased the application of control behaviours that aid in ineffective self-management practices. This reduced the high group’s self-efficacy and motivation to react to the self-management requirements. Savioni and Triberti (2020) found that helplessness led individuals to view the management of their chronic condition as uncontrollable, unpredictable, and unchallengeable, which translated into their daily functioning.

The low HbA1c group redirected their focus to what was in their control, which cultivated a sense of control, fostered autonomy, and reduced the negative appraisal of the condition. Consequently, participants could reduce the severity of T2DM’s impact on everyday life and promote sustained self-regulation of self-management behaviours. Jiraporncharoen et al. (2020) found that the cognitive appraisal of T2DM as incurable but manageable enhanced the participants’ understanding of adherence and facilitated health-related decision-making.

It appears that for CBT interventions to be effective, they must include strategies for cognitive restructuring to challenge and reframe negative thoughts, fostering a more positive and proactive approach (Alyami et al., 2021; Roy et al., 2020). Furthermore, a review by Visagie et al. (2023) indicated that the first step was for individuals to develop cognitive awareness, enabling individuals to identify self-management-related thoughts. This then led to the process of restructuring. The results showed several cognitive restructurings in the low group, which led to a sense of agency. This is an important consideration when looking at which cognitive components to utilise in a CBT intervention. Techniques that enhance self-efficacy, as demonstrated by participants and supported by research, include cultivating a sense of mastery by identifying controllable aspects of their diabetes management, employing problem-solving skills to address challenges proactively (Li et al., 2023; Velázquez-Jurado et al., 2023; Visagie et al., 2023).

### Theme 3: Practising acceptance

The process of altering cognitive appraisals has been closely linked to the acceptance of a chronic condition (Gao et al., 2022). As participants started to acknowledge their condition, both groups mentioned the need for acceptance; however, participants from the low HbA1C group actively engaged with the different aspects they needed to accept. Hörnsten et al. (2011) explained that receiving a diagnosis and accepting the condition are two distinctive processes. This aligns with research by Zalewska et al. (2007) that defined acceptance as an active process where the individual engages with the realities of receiving a diagnosis. The participants from the low group displayed a readiness to cope with the imposed restrictions. They developed a tolerance to the negative appraisals of the condition while integrating the consequences into their everyday routines. Lewko et al. (2007) found that through the process of acceptance, individuals who effectively managed their T2DM had a more controlled response to negative emotions and T2DM itself, whereas feeling limited by the condition inhibited the process of acceptance. A sense of empowerment followed the process of acceptance, as participants from the low HbA1c group were able to identify strategies to effectively self-manage their T2DM. The process of acceptance links to the principles of Acceptance and commitment therapy (ACT), a form of CBT that uses acceptance, mindfulness and behaviour change to improve psychological flexibility (Lean et al., 2018). Visagie et al. (2023) identified that cognitive acceptance enables individuals to disengage from distressing thoughts as the focus is on changing and mediating the relationship that individuals have with their thoughts rather than restructuring the content of their cognitions. This could be an essential component to include in CBT interventions aimed at self-management. A systematic review conducted by Sakamoto et al. (2022) found that ACT could enhance self-care skills and lower glycated haemoglobin levels.

### Theme 4: The mechanisms of behavioural change

The findings on the mechanisms of behavioural change were influenced by their motivation and the resources the participants utilised. Notably, the low HbA1c group demonstrated behaviours that facilitated improved self-management, such as self-directed research, experimentation with healthier alternatives, and understanding their bodies’ cues. In contrast, the high HbA1c group struggled with maintaining new behaviours and often reverted to old patterns. This difference was partly due to the sources of motivation. The high HbA1c group relied more on external motivation, while the low HbA1c group was driven by intrinsic motivation. Walker et al. (2022) found that participants often mentioned external sources of motivation, with little focus on intrinsic motivation. Improved health has been viewed as an intrinsic goal and is associated with autonomy and increased responsibility, as Gunnell et al. (2014) and Sebire et al. (2018) reported. For CBT interventions to be effective, they need to focus on enhancing intrinsic motivation and fostering a sense of personal responsibility. The study revealed that responsibility resulted from intrinsic health beliefs that influenced the participants’ view of self-management, influencing their motivation to act and make changes. Research has reported that when individuals accept responsibility, they increase their knowledge about self-management and engage in effective self-management practices (Adu et al., 2019; Jonker et al., 2018). Motivation facilitates behavioural change. Techniques should include goal-setting exercises that align with patients’ values and intrinsic goals and help individuals navigate internal motivations for self-management.

Behavioural activities to be explored, specifically for T2DM self-management, include fostering autonomous behaviour through self-directed research, making small changes, experimentation, and identifying healthier alternatives. Mamykina et al. (2016) and Tanenbaum et al. (2015) reported similar behaviours that enabled individuals to take ownership of their T2DM. Engaging in these behaviours enabled participants in the current study to make small and gradual changes and develop routines where self-management practices formed part of their lifestyle choices instead of an added burden that needed to be considered. Moreover, creating routines and integrating self-management practices into daily life can help sustain behaviour changes, as consistency is crucial for long-term adherence (Rigby et al., 2022; Rise et al., 2013; Zhou et al., 2015).

Finally, CBT-based interventions should also address the role of social support, emphasising the balance between external support and internal motivation. Social support has been found to positively impact self-management, as expressed by participants in the current study and supported by findings by Al-Dwaikat et al. (2021), Ramkisson et al. (2017), and Rigby et al. (2022). For the high HbA1c group, support appeared to help participants engage in self-management behaviours but did not aid participants in sustaining self-management practices. Participants in the low HbA1c group used a combination of both external and internal resources. Rise et al. (2013) reported that obtaining knowledge and receiving education does not automatically result in adherence to self-management. While social support can be beneficial, it is important to ensure that individuals do not become overly dependent on external resources. Interventions should focus on helping individuals build a supportive network while simultaneously developing their autonomy and self-reliance.

The study’s findings underscore crucial considerations for developing effective CBT interventions. Participants’ cognitive appraisals significantly influenced their emotional experiences and self-management behaviours, highlighting the interplay between thoughts, emotions and behaviours. The strategies employed by the participants align with research indicating the importance of cognitive appraisal and restructuring in the self-management of a chronic condition, emphasising the need for interventions that build individuals’ sense of control, self-efficacy, and positive cognitive appraisals (Fiqri et al., 2022; Vlachou et al., 2022).

## Limitations, strengths, and suggestions for further research

Limitations pertain to the participants’ cultural variability, age range, and socio-demographic backgrounds. In addition, the qualitative nature of the study leads to longitudinal difficulties and difficulty in comparing across studies. The participants' sample size was diverse and included participants from various ethnicities and educational backgrounds; however, greater homogeneous and heterogeneous samples are required to further investigate how these factors influence experiences and perspectives. Additionally, the study’s age range limitation should be considered. Individuals diagnosed at different life stages face different health-related challenges that require further investigation. The setting and qualitative nature of the study imply that the results are context-specific, and the transferability of these results might be limited. Comparing and synthesising findings across different studies can be complex.

These limitations lay the foundations for further studies. Future research can expand on the socio-demographic variation of future samples to incorporate both developed and rural areas and investigate the self-management experiences and requirements within these groups. Sample sizes should include a larger number of participants and focus on different samples based on demographics, such as age, gender, and socio-demographic status.

Longitudinal and experimental studies are necessary to confirm the content of the thoughts, emotions, and behaviours related to self-management and specifically to understand how individuals conceptualise their condition and the processes that are involved in committing to making lifestyle changes and engaging in effective self-management.

## Conclusion

From the findings of the current research study, it was evident that the differences in self-management approaches were how individuals navigated through an intricate framework of subjective experiential facets, which included emotions, cognitions, behavioural, and conative aspects. The findings indicated that the low group could construct a belief system about their diagnosis, abilities, and practices that promoted self-management. The high group displayed a range of negative thoughts, ultimately influencing emotional regulation and limiting health-related behaviours. The notion is that thoughts underlie emotional responses and behavioural reactions. Therefore, it was fundamental to understand the cognitive appraisal and thought patterns of the low and high groups to gauge how this affected their affective state and behavioural practices related to self-management.
